# Atypical Teratoid Rhabdoid Tumor: Proposal of a Diagnostic Pathway Based on Clinical Features and Neuroimaging Findings

**DOI:** 10.3390/diagnostics13030475

**Published:** 2023-01-28

**Authors:** Rosalinda Calandrelli, Luca Massimi, Fabio Pilato, Tommaso Verdolotti, Antonio Ruggiero, Giorgio Attinà, Marco Gessi, Cesare Colosimo

**Affiliations:** 1 Institute of Radiology, Fondazione Policlinico Universitario Agostino Gemelli IRCCS, Largo A. Gemelli, 1, 00168 Rome, Italy; 2 Pediatric Neurosurgery, Neurosurgery Department, Fondazione Policlinico Universitario Agostino Gemelli IRCCS, Largo A. Gemelli, 1, 00168 Rome, Italy; 3 Unit of Neurology, Neurophysiology, Neurobiology, Department of Medicine, Campus Bio-Medico University, 00128 Rome, Italy; 4 UOSD di Oncologia Pediatrica, Dipartimento di Scienze della Salute della Donna, del Bambino e di Sanità Pubblica, Fondazione Policlinico Universitario A. Gemelli IRCCS, Largo A. Gemelli 8, 00168 Rome, Italy; 5 Dipartimento di Scienze della Vita e Sanità Pubblica, Università Cattolica del Sacro Cuore, Largo F.sco Vito 1, 00168 Rome, Italy; 6 Neuropathology Unit, Fondazione Policlinico Universitario “A. Gemelli” IRCCS, Università Cattolica del Sacro Cuore, 00168 Rome, Italy

**Keywords:** atypical teratoid rhabdoid tumor, diagnostic paradigm, MRI, CT

## Abstract

Purpose: To assess the main imaging and clinical features in adult- and pediatric-onset atypical teratoid rhabdoid tumor (ATRT) in order to build a predefined pathway useful for the diagnosis. Methods: We enrolled 11 ATRT patients (10 children, one adult) and we conducted a literature search on PubMed Central using the key terms “adult” or “pediatric” and “atypical teratoid/rhabdoid tumor”. We collected clinical and neuroradiological data reported in previous studies and combined them with those from our case series. A three step process was built to reach diagnosis by identifying the main distinctive clinical and imaging features. Results: Clinical evaluation: neurological symptoms were nonspecific. ATRT was more frequent in children under 3 years of age (7 out of 10 children) and infratentorial localization was reported more frequently in children under the age of 24 months. Midline/off-midline localization was influenced by the age. Imaging findings: Preferential location near the ventricles and liquor spaces and the presence of eccentric cysts were hallmark for ATRT; higher frequency of peripheral cysts was detected in children and in the supratentorial compartment (five out of eight patients with solid-cystic ATRT). Leptomeningeal dissemination at diagnosis was common (5 out of 10 children), while intratumoral hemorrhage, calcifications, and high cellularity were non-specific findings. Histopathological analysis: specific immunohistochemical markers were essential to confirm the diagnosis. Conclusion: In younger children, a bulky, heterogeneous mass with eccentric cystic components and development near ventricles or cisternal spaces may be suggestive of ATRT. ATRT diagnosis is more challenging in adults and relies exclusively on neuropathological examination.

## 1. Introduction

Atypical teratoid rhabdoid tumor (ATRT) is a rare highly malignant embryonal tumor of the central nervous system (CNS), classified as grade four in the 5° edition of the WHO classification for CNS tumors [1,2]. ATRT is mainly reported in the pediatric population, representing 1–3% of all primary CNS tumors and 6.7% in children younger than 3 years of age [3]. In adolescents and adults, ATRT is very rare, with fewer than 60 cases being reported in the current medical literature [4].

ATRT was reported in different locations of the CNS, but it is rare in the cerebellopontine angle cisterns, meninges, cranial nerves, spinal canal, and extradural sites [5,6,7,8].

The diagnostic process for ATRT is challenging because of variable location, histopathologic complexity, the disparity in cytogenetic data, and the lack of specific radiological features.

Often, ATRTs’ imaging features are shared with other embryonal tumors or high-grade lesions especially when they are small or in an unusual site of the brain [9]; however, ATRTs have a worse prognosis due to their particularly aggressive biological nature, their local invasiveness, and their high frequency at disseminating through the cerebrospinal fluid pathways.

Timely diagnosis of ATRTs is critical because these tumors require earlier, more specific, and more aggressive therapy than other high-grade cancers [10]. To date, molecular analysis remains an essential tool for the diagnostic confirmation of ATRTs [8,11].

Herein, we present a case series coupled with a review of the literature to assess the main clinical, pathological, and imaging features of ATRTs in adults and in the pediatric population. Moreover, we propose a diagnostic paradigm with clinical and imaging evaluation useful for recognizing these rare cancers.

## 2. Materials and Methods

### 2.1. Literature Search: Eligibility Criteria and Data Extraction

We searched PUBMED/MEDLINE (until September 2022) using the MeSH-term “adult” or “pediatric” and “atypical teratoid/rhabdoid tumor”. Literature revision was performed independently by two pediatric neuroradiologists and studies were chosen by consensus.

The titles, abstracts, and full texts were reviewed to find potentially eligible articles.

We selected manuscripts written in English of prospective or retrospective studies focusing on the imaging and genetic analysis of ATRT in children and adults. Articles including non-CNS rhabdoid tumors, isolated case reports, unavailable full text, conference, and journal abstracts were excluded. Publications with duplicated information or missing data of interest were further excluded.

Finally, selected papers were included for data extraction and evaluation. The following items were extracted from eligible articles: demographics, tumor location, preoperative imaging features, presence of intracranial and/or spinal metastasis at diagnosis, histopathologic findings, genetic data, surgery, therapy, and length of survival. Some clinical, radiological, and histopathological data were used to generate a diagnostic paradigm and guide the differential diagnosis of ATRT. We considered “distinctive” signs those findings radiologically or clinically relevant for the diagnostic process, while “not specific” signs were those resembling other brain tumors and therefore not peculiar for diagnosis.

### 2.2. Case Collection

At our university hospital, we interrogated our clinical records and found clinical and pathological records, as well as imaging data, from 11 patients treated for ATRT of the CNS. In all patients, the diagnosis was confirmed by neuropathology.

We extracted the following variables: gender, age at presentation, imaging features, extent of surgery (subtotal or gross total resection), complications, adjuvant therapy, and length of survival measured from the time of diagnosis to the date of death or the date of last contact.

The preoperative computed tomography (CT) (HighSpeed and LightSpeed, GE Medical Systems, Milwaukee, WI, USA) and magnetic resonance imaging (MRI) (1.5-T Signa; GE Medical Systems, Milwaukee, WI, USA) were retrospectively reviewed.

For image reading, we applied standardized criteria: localization (supra/infratentorial or midline/off-midline), structure of the origin, maximum size of tumor on the axial plane (in mm), homogeneous versus heterogeneous T2 signal, restriction in diffusion-weighted imaging (signal in DWI/ADC compared with normal brain), presence of peripheral cysts and peritumoral edema (no/minimum/extensive), signs of intratumoral hemorrhage, and bone involvement. After gadolinium administration, enhancement degree (no/minimum/mild/strong and presence of the characteristic “bandlike” pattern) and meningeal dissemination were recorded.

Images were assessed by two pediatric radiologists with 14 years of experience in neuroradiology blinded to pathological diagnosis. Discordances were resolved by consensus.

### 2.3. Statistical Analysis

The dataset was analyzed using descriptive statistics expressed as mean ± SD for continuous variables and as numbers and percentages for categorical variables.

## 3. Results

### 3.1. Medline Review

**Figure 1:**
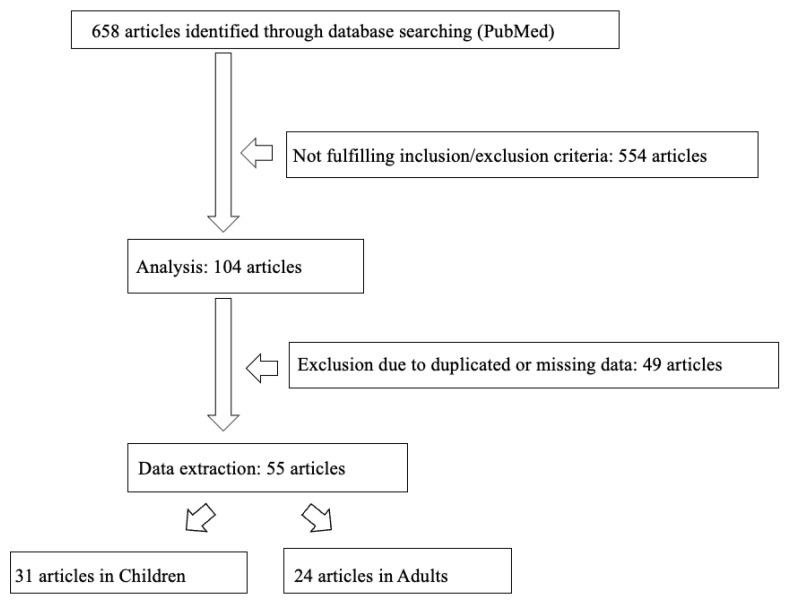
Flowchart of the literature search.

We found 658 articles about ATRT. We excluded 554 articles because they were letters, editorial, isolated case reports, or non-English papers. Moreover, 49 publications were duplicated articles or studies that did not fit with the aim of this review, and they were excluded. A total of 55 articles were selected (31 focused on children and 24 on adults); they covered different topics related to ATRT, such as imaging appearance (children no. 131, adults no. 31), clinical features (children no. 448, adults no. 345), histopathological findings, and genetic mutations (children no. 722, adults no. 111) (Figure 1).

### 3.2. Literature Results

INFANTS/CHILDREN. In previous series about pediatric patients with ATRT, the clinical and histopathological features were described; however, only a few papers have assessed imaging findings [12].

Rorke and Biegel [8] reported that 98% of cases occurred in infants or children and 94% were in patients of 3 years or younger with a male prevalence. Fifty-two percent occurred in the posterior fossa and tended to occur off-midline, 39% in the cerebral hemispheres or suprasellar region, 5% in the pineal region, and 2% in the spinal cord. Location in the posterior fossa was more common in children younger than 24 months of age [13]. Both in infratentorial and supratentorial compartments the location next to the ventricles was frequent [14]. One-third of the ATRT patients had evidence of leptomeningeal dissemination at the time of diagnosis [13,15]. Clinical presentation was consistent with the location of the tumors and the age at the time of diagnosis [13]. Total or near-total resection was achieved in 30% of patients [13].

The treatment for pediatric patients with ATRTs included the attempt to obtain total resection, followed by chemotherapy (ChT) and radiotherapy (RT) whenever the child’s age and clinical status allowed it [8,12,16,17,18]. However, the prognosis of patients with ATRTs remains poor, with reported mean survival rates in the range between 6 and 15 months [8,13,19,20], but long-term survival was also reported in some cases treated with craniospinal radiotherapy [8] or gamma knife radiosurgery on relapse [12]. No improvement in survival was reported for patients treated with high-dose chemotherapy [21,22]. On the other hand, children who did not receive any treatment after surgery died within 1 month [8].

Imaging features of ATRTs have been described in small case series [13,23,24]. The case series involving a larger number of patients (10–17 patients) [15,23,25] reported large, heterogeneous enhancing tumors with eccentric cysts in 70–72% [26] and hemorrhagic areas in 40–60% [24,27], variable T2 signal, restricted signal on DWI, or a high attenuation on CT in the solid parts of the tumor, due to the high cellularity. Compared with infratentorial ATRTs, supratentorial ATRTs showed a higher incidence of cystic components (about 80% vs. 63%) and a higher [25] evidence of thick and wavy enhancing of the wall surrounding the central cyst. Edema occurred more often in supratentorial ATRTs compared with infratentorial tumors [25].

ADULTS. The most complete studies in adult ATRT patients were by Broggi et al. [28] and Chan et al. [29] and were focused on clinicopathologic features, therapies, and outcomes. The average age at diagnosis was 38.3 years with a range from 18 to 80 years and prevalence in females [28]. Signs and symptoms were mainly related to the location of the tumor. The most common intracranial location was the sellar region (46%), followed by the cerebral hemispheres (21.9–32%); other uncommon locations were the pineal region (6–8%), cerebellopontine (CP) angle (4–6%), cerebellum (2–4%), tectum (1%), trigeminal nerve (1%), thalamus (1%), and intraventricular localization (1%). Midline tumors occurred mainly in adult patients over 40 years, while laterally located tumors were more prevalent in patients younger than 40 years. At diagnosis, a few patients (14–31.3%) had dissemination of disease and local invasion [28,29]. The treatment regimen always followed gross total resection (GTR in 29%), subtotal or partial resection (46.9%), or biopsy (3.1%). The majority of adult patients (56–57.3%) received combined radiotherapy and chemotherapy, while the remaining adult patients received radiotherapy alone (13.5–16%), chemotherapy alone (1–2%), stereotactic radiosurgery alone (2.1–4%), or did not receive adjuvant therapy (8.3%). Between 51% and 62% of patients died of their disease with a mean time to death of about 17 months, but patients receiving radiotherapy and chemotherapy had longer survival than either patients treated with radiotherapy alone or patients who did not receive adjuvant therapy.

Radiological findings of ATRTs were derived mainly by small case series [30,31] and only a few reviews are available [29,32,33].

Kanoto et al. [33] described neuroimaging in 38 adult patients with ATRTs and 21 were in the cerebrum compartment. Most of the cases showed hyperattenuation on CT (91%), hypointensity on T1-weighted resonance imaging (80%), mixed-signal on T2-weighted imaging (72%), restricted diffusion of the solid component (100%), and heterogeneous enhancement (70%); 56% of cases had cysts/necrosis, 42% had hemorrhages, and 33% had calcifications. A thick and irregular parietal enhancement surrounding the cystic or necrotic component of the lesion was frequent in the supratentorial compartment, but was not specific for adult ATRTs [19]. Mild edema was described frequently in adult-onset ATRTs [33,34]. Another study reported neuroimaging findings in sellar ATRT cases, reporting overlapping features between sellar ATRTs and pituitary macroadenomas; in 44.4% of cases cavernous sinus invasion and in 22.2% cystic portions of the tumor were described [32].

### 3.3. Case Series Findings

The clinical characteristics of the enrolled patients are shown in Table 1. There were three females and eight males. The age of presentation in the pediatric population ranged between 3 days and 10 years (average age of presentation was 28.62 ± 36.18 months). The only adult patient was 43 years old.

The most common symptoms at presentation were related to increased intracranial pressure and included macrocephaly (no. five patients), vomiting (no. three patients), headache (no. two patients), ptosis (no. one patient), strabismus (no. one patient), diplopia (no. one patient), and seizure (no. two patients). Six patients had acute hydrocephalus at the time of presentation.

Unenhanced CT (NECT) was performed in five patients while MRI was performed in all patients.

Four tumors were in the posterior fossa (floor of IV ventricle) and seven were in the supratentorial compartment (frontal lobe no. one, frontotemporal lobes no. one, temporal lobe no. two, temporo-occipital lobes no. one, parieto-occipital lobes no. one, sellar-suprasellar region no. one). There was a particular left lateralization in the supratentorial compartment except for the only adult whose sellar/suprasellar lesion had midline location. On the other hand, all infratentorial tumors involved the floor of IV ventricle and grew on midline. Only three out of four SMARCB1 mutated ATRTs located in PCF were classified according to DNA methylation profiling (two ATRT-tyrosine and one ATRT- sonic hedgehog tumors) (Table 1 and Table 2).

**Figure 2:**
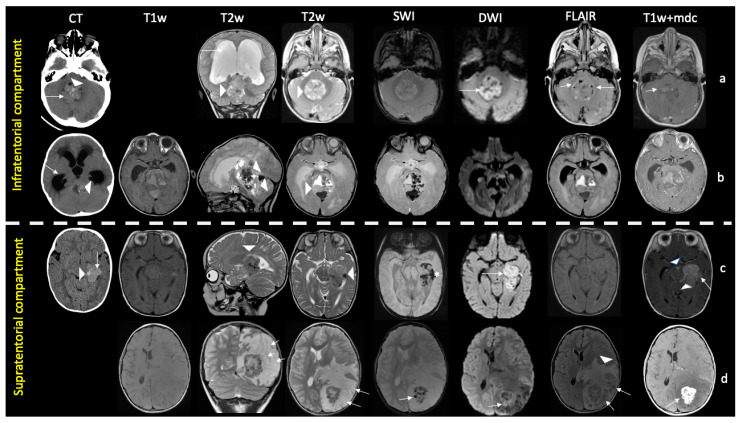
Imaging findings of pediatric ATRT in supra- and infratentorial compartments. Infratentorial ATRTs (rows (**a**,**b**)). Row (**a**): One-year-old child with a prevalently solid mass centered in the IV ventricle, hyperintense to gray matter (arrowheads in T2w) with high density/restricted diffusion (arrows in CT, DWI) due to hypercellularity, minute eccentric cystic components (arrows in FLAIR), and mild enhancement (arrow in T1+mdc). Intratumoral foci of Ca++ (arrowhead in CT) are present, interstitial edema is absent. Hydrocephalus is shown (white arrow in T2). Row *(***b**): a 4-month-old child with a bulky heterogeneous mix mass with epicenter in the posterior cranial fossa (IV ventricle and vermis) and supratentorial extension (arrowheads in T2w). The lesion contains small hyperdense foci of Ca++ (arrowhead in CT), hemorrhage both in solid and cystic component (hyperintense signal in T1w, hyperintense and hypointense signal in T2w, hypointense signal in SWI). Interstitial edema is absent. Hydrocephalus is detected (white arrow in CT). Supratentorial ATRTs (rows (**c**,**d**)). Row (**c**): a 7-month-old child with a solid and heterogeneous intracranial mass centered in the temporal lobe, near to lateral ventricle (arrowheads in T2w). It shows high density (arrow in CT) and restricted diffusivity (arrow in DWI) due to hypercellularity, containing foci of Ca++ (arrowhead in CT), areas of hemorrhage (* in SWI), and marked enhancement (arrow in T1+mdc). Note widespread subarachnoid metastatic disease filling the interpeduncular and perimesencephalic cisterns (arrowheads in T1+mdc). Row *(***d**): a 10-year-old child with a mixed lesion in the parietal lobe consisting of a heterogeneous solid component due to hypercellular (arrow in DWI) and hemorrhagic areas (arrow in SWI) and multiple eccentric cysts (arrows in T2 and FLAIR); note marked enhancement (arrow in T1+mdc) of the solid component and abundant perimarginal edema (arrowhead in FLAIR).

ATRTs in pediatric patients were large at the diagnosis (average 50 × 40.3 mm). Four tumors were predominantly solid (two in posterior cranial fossa, one in temporal lobe, one in fronto-temporal lobes), while six were heterogeneous tumors with solid and cystic components. Almost all cysts were peripherally located and more frequent in the supratentorial compartment, five of our cases (50%) had calcifications, and four (40%) had intratumoral hemorrhage. Perifocal edema varied but was more frequent in the supratentorial compartment than in the infratentorial compartment, ranging from minimum to extensive. The solid part of these tumors showed restricted diffusion compared with normal brain parenchyma in nine patients, (hyperintensity on DWI and hypo-intensity in ADC), indicating high cellularity of the tumor. Contrast enhancement was noted in nine patients (one supratentorial tumor showed a “wavy” bandlike pattern) while in only one patient, tumor in PCF did not show any enhancement (Figure 2).

**Figure 3:**
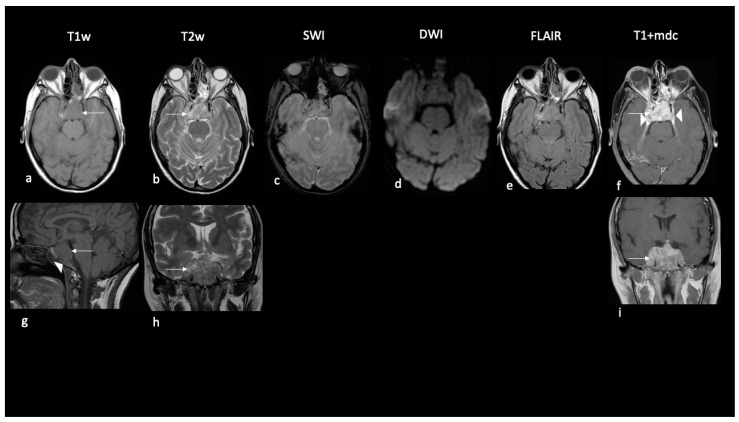
Imaging findings of sellar ATRT in an adult. Ax T1w (**a**,**g**); Ax T2w (**b**,**h**); SWI (**c**); Ax DWI (**d**); Ax FLAIR (**e**); Ax T1w+mdc (**f**,**i**). A local aggressive mass centered in the sellar region in an adult of 43 years. The mass is prevalently solid, hypointense in T1w (arrows in (**a**,**g**)) and hyperintense in T2w (arrows in (**b**,**h**)) to gray matter with marked enhancement (arrow in (**f**,**i**)) invading cavernous sinus (arrowheads in (**f**)) and clivus (arrowhead in (**g**)). Decreased diffusivity and interstitial edema are absent.

The adult-onset sellar-suprasellar tumor was predominantly solid and homogeneous and did not show restricted signal on DWI or edema but showed destruction of the clivus bone (Table 2, Figure 3).

**Figure 4:**
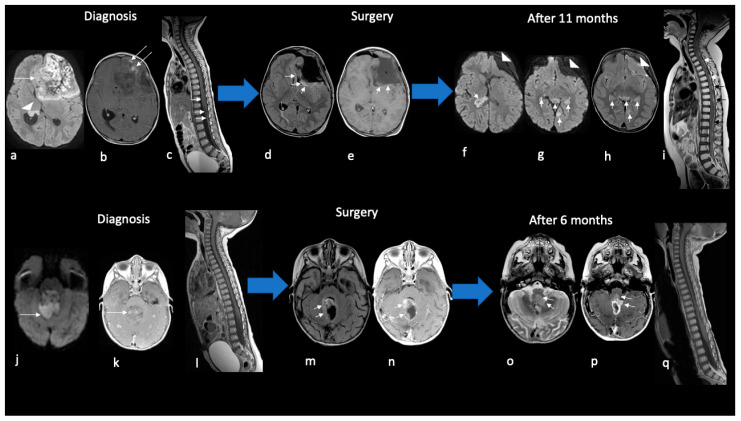
Imaging findings of disease progression in children with ATRTs. Ax DWI (**a**,**f**,**g**,**j**); Ax T1w (**e**); Ax T1w+mdc (**b**,**k**,**n**,**p**); Sag T1+mdc (**c**,**i**,**l**,**q**); Ax FLAIR (**d**,**h**,**m**); Ax T2w (**o**). An about 2-year-old child with frontal ATRT (**a**–**i**). At diagnosis, axial DWI MR shows reduced diffusivity of ATRT at the primary site in the frontal lobe (arrow in (**a**)) and at metastases in the lateral ventricle (arrowhead in (**a**)); mild enhancement is present (arrows in (**b**)). Sagittal T1+mdc MR of the spinal column shows leptomeningeal enhancement along the spinal cord (arrows in (**c**)). Gross total resection was performed after 10 days showing a large surgical cavity with parenchymal reactive alterations but without apparent local disease residue (arrows in (**d**,**e**)). After 7 months from surgery, the surgical cavity is collapsed (arrowheads in (**f**–**h**)); note the increase of both intracranial metastases in ventricles and perimesencephalic cistern (arrows in (**f**–**h**)) and spinal dissemination with nodular leptomeningeal foci (arrows in (**i**)). The patient died after 20 months. An about 1-year-old child with posterior cranial fossa ATRT (**j**–**q**). At diagnosis, axial DWI MR shows reduced diffusivity of ATRT centered in the cerebellar vermis and filling the IV ventricle (arrow in (**j**)); mild enhancement is detected after gadolinium (arrow in (**k**)) but neither intracranial nor spinal metastases are noted. Gross total resection was performed after 6 days showing the surgical cavity margined by parenchymal reactive alterations (arrows in (**m**,**n**)) without local disease residue. After 4 months from surgery local recurrence appears along the left anterolateral border of the surgical cavity (arrows in (**o**–**p**)) while no spinal dissemination is detected. The patient died after 9 months.

In four pediatric patients (40%), tumors showed leptomeningeal spreading at diagnosis, either in intracranial cerebrospinal fluid (CSF) or in intracranial and spinal CSF (Figure 4).

Gross total resection was achieved in four pediatric patients, while subtotal resection was achieved in seven patients (six children and one adult). Ten out of eleven patients received chemotherapy, of which three patients also had high-dose chemotherapy and autologous hematopoietic stem-cell transplantation (two pediatric and one adult); only two patients also received RT (one child and one adult). Most of the patients died in four–nine months, while those patients treated with high-dose chemotherapy and radiotherapy had a longer outcome after diagnosis, although one of them was in disease progression. The only pediatric patient who did not receive therapy died after 20 days (Table 1, Figure 4).

## 4. Discussion

**Figure 5:**
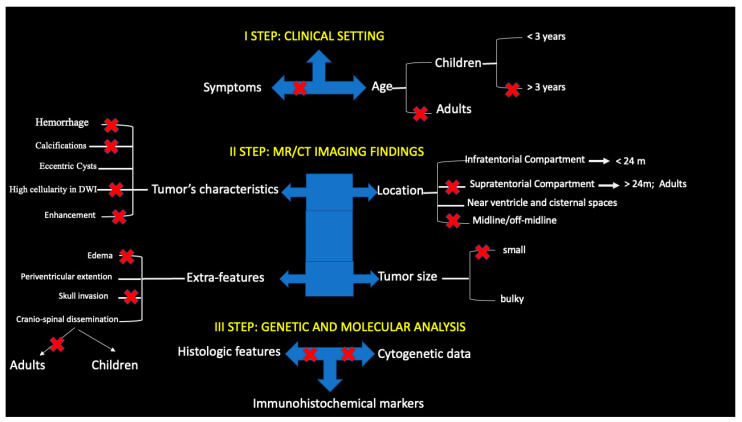
A flowchart of clinical and imaging findings in ATRT. A three-step route shows distinctive features of ATRT able to guide the ATRT diagnosis. **X** indicates nonspecific findings.

ATRT is a rare pediatric tumor, and it is even more rare in adults. Because imaging characteristics of ATRTs may overlap with other CNS tumors, preoperative diagnosis of ATRTs are a challenge and they are often misdiagnosed. The available clinical literature mainly consists of reviews of case series reporting clinical features and outcomes both in pediatric and adult ATRTs, while the available radiological literature consists of isolated case reports and small case series. By combining the available literature with the radiological and clinical findings of our case series, we built a stepwise paradigm for ATRT diagnosis, capable of improving radiological recognition of this rare cancer (Figure 5).

### 4.1. First Step: Clinical Setting

The clinical symptoms are nonspecific because they mainly depend on the location of the tumor and its mass effect on the CNS [35]. As also confirmed in our series, the most commonly reported symptoms are nausea and vomiting as the result of increased intracranial pressure, while ataxia and gait disturbance may be present in children over 2 years of age with cerebellar involvement. VI and VII cranial nerve palsies are usually associated with tumors invading the cerebellum-pontine angle, while headache and diplopia are found in those rare cases of sellar/suprasellar ATRTs [36,37,38,39].

Age. Although rare, ATRTs are mainly found in infants and in children below 3 years of age [8,20], as confirmed by our cases; on the other hand, ATRTs in adolescents and adult patients are rare, with an age distribution at the time of diagnosis ranging between 18 and 69 years and with a median age of about 32 years [29,40,41]. In the pediatric age, patients with ATRTs had a male predominance up to the age of 3 years, which then seemed to disappear [3]; in adults, male gender is more prevalent between the age of 18 and 40 years and is more often associated with non-midline tumors, while female predominance is notable for midline tumors, including those of sellar region [42,43].

### 4.2. Second Step: MR/CT Imaging Findings

The radiologic assessment is crucial for the pre-operative diagnosis of ATRT. To effectively reach a diagnosis, a standardized approach using CT and MRI should be taken into account by considering several features.

Locations. ATRT may arise in either the supratentorial or infratentorial areas [14]; often, this tumor tends to grow up along the ventricles and into the adjacent cisternal space, to involve the ventricular system by direct extension, and to occur off-midline [13,14]. There are discrepancies among studies with regard to the predilected site, whether supra- or infratentorial compartment, probably because of the relatively small number of patients included in each study [14]. One of the most extensive reviews by Oka and Scheithauer described that the posterior fossa includes 61% of ATRT locations with a predilection for the cerebellum; followed by the cerebral hemisphere (20%) with the frontal lobe as the most common location; the suprasellar/third ventricular region (5%); and, rarely, the spinal cord (1%) [44]. Other series reported a similar proportion of infratentorial and supratentorial ATRTs [45] or a higher prevalence of supratentorial ATRTs, as we found in our case series. Some studies reported that the primary location in the posterior fossa is more common in children younger than 24 months of age (>80%) than in older children [13]. Our case series confirmed that the predominant location of ATRT in the brain may depend on age and/or molecular subgroup [26].

In the posterior fossa, ATRTs are usually located around the floor of the fourth ventricle and three subgroups corresponding to the region of origin from the fourth ventricle were reported: the superior medullary velum (SMV), cerebellopontine angle (CPA), and inferior medullary velum (IMV) [14]. Some studies related the tumor location to the molecular analysis defined by gene expression and/or DNA methylation profiling and classified pediatric ATRTs in three subgroups: ATRT–myelocytomatosis oncogene [MYC] tumors predominantly occurred in the supratentorial compartment and in patients older than 3 years (median age 27 months), ATRT-tyrosine [TYR] tumors occurred preferentially in infratentorial locations and in patients younger than 1 year (median age 12 months), and ATRT–sonic hedgehog (SHH) tumors occurred both at the supra- and infratentorial compartment and represented an intermediate subgroup between younger ATRT-MYC patients and older ATRT-TYR patients (median age 20 months) [26,28,46]. Although the off-midline location has been reported to be a characteristic of infratentorial ATRT-TYR, some studies found 50% of midline tumors (cerebellar vermis, fourth ventricle) in infratentorial ATRT-TYR, suggesting that the midline/off-midline location is not a subgroup-specific feature [26]. The latter hypothesis seems to be confirmed by our series of cases in which two out of three posterior cranial fossa-onset ATRTs detected in children under 12 months of age were midline ATRT-TYR tumors.

In adults, rhabdoid tumors are frequently supratentorial, whereas the most common intracranial location is suprasellar (46%), followed by the cerebral hemispheres (32%), while only rare cases are in the cerebellum and in the spinal cord [47]. Johann et al. and Alzoubi et al. proved that the methylation profile of rare cases of adult ATRTs matched with pediatric ATRTs of the MYC subgroup [43,48].

Considering both adults and children, very rare cases of ATRTs were detected in extra-axial locations including the intraventricular site and cerebellopontine angle. Other cases originated from the cranial nerves, brainstem, and spinal cord or primarily affected the leptomeninges; in the latter case, the tumor is termed as primary diffuse leptomeningeal ATRT [6,7,49,50]. In these rare cases of ATRTs, when not accompanied by peculiar imaging features, the diagnosis may be delayed.

Tumor size. It has been reported that the tumor’s volume at diagnosis is related to the supra- or infratentorial localization rather than to molecular subgroups characteristics [26]. Accordingly, we found larger sizes of supratentorial ATRTs than infratentorial ones [27]; this may be due to the greater accommodation and later onset of neurological symptoms of supratentorial tumors compared with infratentorial ones [51].

Tumor’s characteristics. Several aspecific imaging features such as intratumoral hemorrhage, calcifications, cysts, high cellularity of the solid component, and heterogenous enhancement have been reported [13,23,25,51,52], although some peculiar imaging findings may be helpful for the diagnosis; moreover, differences may be identified both about the supra/infratentorial location and the subgroups to which ATRT belongs. The presence of eccentric cysts has been recognized as suggestive of ATRT; the frequency of peripheral cysts does not depend on the supra- or infratentorial localization [26,27], but it is mainly influenced by the molecular subgroup and, interestingly, eccentric cysts ranged from only 40% in ATRT-MYC to 71% in ATRT-SHH and even 94% in the ATRT-TYR group [26]. On the other hand, peritumoral edema is not a peculiar feature and it seems to be not related to the molecular subgroups but to its localization [26]. Accordingly, our data show that supratentorial ATRTs have more surrounding edema than infratentorial tumors do because they had more available space to grow in, becoming symptomatic at the later stages [31]. Additionally, the enhancement pattern is not peculiar for ATRTs ranging from no or minimal enhancement to strong enhancement. Some studies reported that the type or degree of enhancement is related to the molecular subgroup; in the infratentorial compartment, pediatric ATRTs in the SMV area may have no or minimal enhancement compared with CPA and IMV areas [14], while in the supratentorial compartment a distinct and unusual pattern of a “wavy” bandlike of strong enhancement surrounding, completely or partially, a central cystic or necrotic area may be detected both in pediatric and adult supratentorial ATRT-TYR tumors [27,46].

In our case series, one pediatric supratentorial ATRT showed a “wavy” bandlike pattern and one pediatric infratentorial ATRT had no enhancement; however, the lack of molecular analysis in all our patients does not allow us to evaluate the relationship between enhancement and the molecular subgroup.

Leptomeningeal dissemination detected by contrast enhancement imaging is common in ATRTs and may guide the diagnosis; the proximity to the ventricles could explain the frequent leptomeningeal dissemination of these tumors, reflecting the extremely aggressive nature of these tumors [25]. In our case series, tumor dissemination was observed in one-third of patients at diagnosis and seems to be more frequent in the infratentorial compartment in children, rather than in the supratentorial compartment in adults [25,40,53]. Considering leptomeningeal dissemination, no significant difference has been reported among different molecular subgroups [26]; on the other hand, some authors reported that SMV-originating ATRTs had scarcer leptomeningeal dissemination at presentation than those tumors originating from CPA and IMV regions [14]. The low rate of tumor dissemination may be related to the lack of tumor enhancement in SMV-ATRT, which indicates an intact tumor–vessel permeability preventing the easy spreading of tumor cells. However, Tomita and Frassanito reported that all their patients with an SMV-originating ATRT died within months of diagnosis, indicating that scarce seeding at presentation does not indicate favorable biological behavior of the tumor [54].

Bone involvement [13,55] and extracranial metastases including lung [56,57] are rarely reported and do not constitute a hallmark of ATRTs. Tumors in the sellar/suprasellar region are the ones that show a more frequent local aggressive behavior because they often invade the cavernous sinus, clivus, and other important structures and they cannot be removed completely [32]; we found this behavior in our series as well.

Differential diagnosis. Often, the variable clinical presentation, but also some radiologic and histologic features, make ATRTs hard to discriminate from other brain tumors. When they are in the infratentorial compartment, they can be misdiagnosed as MB or anaplastic ependymomas, while when they are in the supratentorial compartment they may resemble some embryonal tumors, such as embryonal tumor with multilayered rosettes (ETMR) or high grade gliomas and anaplastic ependymomas [24,39,58,59,60,61,62,63].

ATRT shares some features with the above-mentioned CNS tumors. The restricted diffusion of the solid component due to the high cellularity is a feature found also in MB, ETMR, and supratentorial high-grade gliomas; when ATRT has an infratentorial location, the low frequency of intratumoral calcifications and hemorrhage is also a common feature of MB [64,65].

The tendency to occur off-midline and the presence of intratumoral calcifications and hemorrhage are differential diagnostic criteria for anaplastic ependymomas; the high rates of cystic components, calcifications, and hemorrhage, together with a heterogeneously enhancing wall surrounding a central cystic region, represent features for differential diagnosis with other embryonal tumors [66].

However, in pediatric ATRTs, the presence of a large tumor with eccentric cysts and growing near ventricle or cisternal spaces, combined with some other features such as a very young age and the tendency to disseminate at diagnosis, remain hallmarks [13] and help to formulate a correct neuroradiological differential diagnosis [25].

If the diagnosis of ATRT may be easily suspected in very young infants, in adults the differential diagnosis is more challenging and, often, the correct diagnosis is guided by the histopathological result. ATRT often mimics, in terms of clinical features and image findings, high-grade glioma, pineoblastoma, germinoma, pituitary macroadenoma, and other embryonal tumors such as embryonal tumor with multilayered rosettes [48]. In fact, all of these tumors share common sites, the presence of hemorrhage, calcifications, high DWI signal, enhancement features, and, in cases of sellar location, the invasion of the cavernous sinus and clivus [31,32].

### 4.3. Third Step: Genetic and Molecular Analysis

Because the MRI findings of ATRT often overlap with other pediatric high grade brain tumors, and their clinical outcomes are different, the pathological confirmation of ATRT is mandatory.

The disparity in cytogenetic data involving chromosome 22 and, rarely, the chromosomes 6 and 11 [23,67,68], reflects the morphological heterogeneity of these tumors [15]. According to the previous literature, abnormalities involving chromosome 22 typically result, in 70% of cases, in loss of the hSNF5/INI1/SMRCB1/BAF47 gene and, in extremely rare cases, in loss of the BRG1/SMARCA4 gene [23,67,68]. To date, the diagnosis confirmation of ATRT requires both recognition of alterations in SMARCB1/SMARCA4 genes and specific immunohistochemical markers recognition expressed by rhabdoid cells such as epithelial membrane antigen (EMA), vimentin, smooth-muscle actin (SMA), and loss of nuclear expression of INI-1 protein [69].

Gene sequencing and DNA methylation analyses are some of the most powerful tools currently available to assess biological features of the tumor and identify therapeutic targets.

Studies demonstrated that mutations of SMARCB1 may be found in all domains of the protein, but many mutations affect the C-terminal region, and truncating C-terminal SMARCB1 mutations are associated with a relatively favorable outcome [70,71,72]. On the other hand, SMARCA4-mutated ATRTs show a more aggressive behavior and they are associated with a higher frequency of inherited germline mutations, younger age, and worse prognosis compared with SMARCB1 [70,73].

Other studies based on DNA methylation profiles demonstrated that the three distinct molecular subgroups (ATRT-TYR, ATRT-SHH, and ATRT-MYC) differ in terms of age at diagnosis, tumor location, type of SMARCB1 alterations, and overall survival. In particular, ATRT-TYR, especially in newborns older than 12 months, have been associated with a slightly better prognosis compared with the other subgroups [70].

In agreement with previous studies, our data demonstrated a shorter survival in patients with SMARCA4-mutated tumors than those with SMARCB1-mutated tumors, confirming the biologically more aggressive nature of SMARCA4-mutated tumors. Unfortunately, DNA-methylation profiling was performed in only a few of our patients, while gene sequencing was unavailable. Thus, we cannot provide a combined reading of histopathological data together with neuroimaging findings. Further studies based on gene sequencing and DNA-methylation profiling are warranted to address the relationship between histopathologic profiles and radiologic features.

Currently, no specific guidelines have been established for the management of ATRTs, and a multimodal approach including gross surgical resection coupled with chemotherapy (ChT) and radiotherapy (RT) according to the child’s age and clinical status [8,12,16,17,18] is the preferred treatment modality. Although radiation therapy is an important component of therapy because it has a positive impact on survival [52,74], it is usually avoided in patients younger than 3 years of age due to long-term neurocognitive sequelae [75].

To date, the prognosis for ATRTs remains poor, especially in patients younger than 3 years of age and in the presence of a residual tumor or metastasis [16,23,76,77,78,79]. In pediatric populations, life expectancy is shorter than in adults, with a survival time that averages 15 months in children and 38 months in adults [31,78,80], suggesting that adult ATRTs have a different biological nature or simply that adults may better tolerate aggressive adjuvant treatments than young children [40,53,77]. A much longer survival is reported in a subset of children treated with radical surgery and aggressive chemotherapy and in children that had at least received local radiotherapy [11,81]. Moreover, prolonged survival of more than 6 years has also been reported with a multimodality approach of surgical resection, triple intrathecal chemotherapy, and gamma knife radiosurgery by Hirth et al. [12].

In our series, all but one of our pediatric patients received therapy after diagnosis; two children over 21 months of age who received high dose ChT and intrathecal ChT, associated or not with RT, had a longer survival or a complete remission of the disease. Additionally, the adult patient who received combined ChT and RT had complete remission of the disease. Although reluctance exists in using radiotherapy in young patients because of the risk of functional impairment of the developing brain [82,83], our data seem to confirm that aggressive chemotherapy and radiation therapy might improve the outcome. In the near future, the availability of gene sequencing might be a promising ground for the development of targeted and personalized therapies.

Our diagnostic paradigm, based on literature data, may be a useful and practical tool for the differential diagnosis of ATRTs, but the lack of statistical support in selecting some “distinctive” radiologic and clinical findings is a limitation and some possible bias may not be ruled out; future studies are warranted to validate this diagnostic process.

## 5. Conclusions

The rarity of ATRTs occurring in the pediatric and adult population, along with their unusual cytology and lack of specific radiological features, make their diagnosis a challenge. However, especially in childhood, the very early age of onset, together with the predilection for the development near cistern spaces or ventriculi and the presence of eccentric cysts, should make the radiologist aware of the possibility of ATRT. On the other hand, in adult patients, the diagnosis remains a challenge and only the histopathological analysis allows us to make the correct diagnosis. A multidisciplinary approach involving radiologists, neurosurgeons, and pathologists through sharing clinical and pathological information may improve diagnostic accuracy, speeding up the diagnostic process and allowing prompt therapy.

## Figures and Tables

**Table 1:** Clinical features and outcome in 11 patients with atypical teratoid rhabdoid tumors.

Pt	Age at Diagnosis	Gender	Immunostains	Genetic Mutation (Molecular Analysis)	Presenting Features	Hydrocephalus	Surgery, Therapy	Remission/Progression	/Survival
1	3 d	M	Vimentina, EMA, Pan-CK e GFAP	BRG1/SMARCA4	Intracranial hypertension, macrocephaly	yes	STR, CT (2 cycles ICE and 2 cycles CAV)	Progression	EOL (after 5 months)
2	1y 4m	M	Vimentina, SMA EMA, Pan-CK e GFAP	BRG1/SMARCA4	Intracranial hypertension, macrocephaly	yes	STR, CT (2 cycles Carboplatin and VP16)	Progression	EOL (after 4 months)
3	4 m	M	SMA, vimentina	INI1/SMARCB1 (ARTR-TYR)	Intracranial hypertension, macrocephaly, vomiting	yes	STR, CT (2 cycles ICE and 2 cycles CAV)	Progression	EOL (after 6 months)
4	7 m	M	GFAP, pan-CK, SMA, vimentina, EMA	BRG1/SMARCA4	Ptosis	no	STR	Progression	EOL (after 20 days)
5	4 d	F	SMA, vimentina, EMA	INI1/SMARCB1 (ATRT-TYR)	Intracranial hypertension, macrocephaly	yes	GTR, CT (3 cycles ICE and 3 cycles CAV)	Progression	EOL (after 9 months)
6	1y 9 m	M	Vimentina, SMA EMA	INI1/SMARCB1	Seizure	no	GTR, CT (3 cycles ICE and 3 cycles CAV), HD-CT (1 cycle Thiotepa-Carboplatin), Autologous haematopoietic stem-cell transplantation	Remission	Alive
7	2y 5m	M	GFAP, MAP2, SMA, vimentina	INI1/SMARCB1	Intracranial hypertension, convergent strabismus, jet vomiting	yes	GTR, CT (2 cycles CAV and 2 cycles ICE and it), RT, HD-CT (one cycle: Thiotepa-Carboplatin, one cycle: Thiotepa), Autologous hematopoietic stem-cell transplantation, CT (3 cycles Temodal)	Progression	Alive (after 20 months)
8	3y 6m	M	Vimentina, SMA EMA, Pan-CK e GFAP	INI1/SMARCB1 (ATRT-SHH)	Intracranial hypertension, macrocephaly, vomiting	yes	STR, CT (2 cycles ICE and 2 cycles CAV)	Progression	EOL (after 5 months)
9	3y 11m	F	GFAP, MAP2, SMA, vimentina	INI1/SMARCB1	Headache	no	GTR, CT (2 cycles ICE and 2 cycles CAV)	Remission	EOL (after 7 months)
10	10y	M	Pan-CK, SMA, vimentina, EMA	INI1/SMARCB1	Seizure	no	STR, CT (2 cycles ICE and 2 cycles CAV)	Progression	
11	43y	F	vimentina, EMA	INI1/SMARCB1	Headache and diplopia	no	STR, CT (3 cycles ICE and 3 cycles CAV), RT, HD-CT (1 cycle: Thiotepa-Carboplatino, one cycle: Thiotepa), Autologous haematopoietic stem-cell transplantation, CT (12 cycles Temodal)	Remission	Alive (after 36 months)

Pt, Patient; ATRT, atypical teratoid rhabdoid tumor; ATRT-TYR, ATRT-tyrosine; ATRT-SHH, ATRT-sonic hedgehog tumors; GTR, gross total resection; STR, subtotal resection; d, day; m, month; M, male; F, female; S, supratentorial; I, infratentorial; CT, chemotherapy; HD-CT, high-dose chemotherapy; ICE, ifosfamide/carboplatin/etoposide; CACV, cyclophosphamide/adriblastine/vincristine; VP16, etoposide; it, intrathecal; EOL, end of life.

**Table 2:** Preoperative imaging findings in 11 patients with atypical teratoid rhabdoid tumors.

Pt	Location Category (Specific Location)	Tumor Size (mm)	Density/Signal (Homogeneous/Heterogeneous)	Composition (Solid/Mix)	Hemorraghe	Ca++	T2 Signal	DWI/ADC	Enhancement/ LM Dissemination	Peritumoral Edema	Bone Invasion
1	S (left temporo-occipital)	50 × 42	Hetero	Mix (peripheric cysts)	yes	yes	Solid:hypo Cysts:hyper	High DWI, low ADC	mild/no	minimum	no
2	I (IV ventricle)	17 × 16	Homo	Solid	-	yes	Solid:hypo	High DWI, low ADC	strong/no	none	no
3	I (IV ventricle)	76 × 41	Hetero	Mix (peripheric cysts)	yes	-	Solid:hypo Cysts:hyper	Iso DWI	none/no	none	no
4	S (left temporal lobe)	43 × 30	Hetero	Solid	-	yes	Solid:hypo	High DWI, low ADC	strong/yes	minimum	no
5	I (IV ventricle)	40 × 39	Hetero	Mix (peripheric cysts)	-	yes	Solid:iso Cysts:hyper	High DWI, low ADC	minimum/no	none	no
6	S (left temporal lobe)	52 × 39	Hetero	Mix (peripheric cysts)	-	-	Solid:iso Cysts:hyper	High DWI, low ADC	mild/no	minimum	no
7	S (left frontal and temporal lobes)	75 × 77	Hetero	Solid	yes	-	Solid:hyper	High DWI, low ADC	minimum/yes	none	no
8	I (IV ventricle)	21 × 27	Hetero	Solid	yes	-	Solid:hyper	High DWI, low ADC	mild/yes	none	no
9	S (left frontal lobe)	55 × 41	Hetero	Mix (peripheric cysts)	-	-	Solid:iso Cysts:hyper	High DWI, low ADC	mild/yes	extensive	no
10	S (left parieto-occipital lobes)	71 × 51	Hetero	Mix (peripheric cysts)	-	yes	Solid:hypo Cysts:hyper	High DWI, low ADC	mild/no	extensive	no
11	S (sellar-suprasellar)	35 × 27	Homo	Solid	yes	-	Solid:hyper	-	strong/no	none	yes

Pt, Patient; S, supratentorial; I, infratentorial; Hetero, heterogeneous; Homo, homogeneous; Ca++, Calcifications; LM, leptomeningeal; iso, isointensity; hypo, hypointensity; hyper, hyperintensity.

## Data Availability

The data that support the findings of this study are available from the corresponding author, [R.C.], upon reasonable request.
